# The Burden of Serious Falls Among Older Adults: Evidence from Hospitalizations in Philadelphia, PA

**DOI:** 10.1093/geroni/igaa057.2254

**Published:** 2020-12-16

**Authors:** Megan Todd, Kevin Pressley, Meagan Pharis

**Affiliations:** Philadelphia Department of Public Health, Philadelphia, Pennsylvania, United States

## Abstract

Falls among older adults cause acute injury, are associated with subsequent mortality, and cost billions of dollars in medical expenses each year. However, research on falls is lacking compared to other causes of morbidity. In our rapidly aging world, a better understanding of the populations at greatest risk is urgently needed. In this paper, we used 2018 data on every inpatient hospitalization in Philadelphia and from the American Community Survey to estimate the prevalence of serious falls among older adults (60+) by age, sex, and race/ethnicity. We further assessed the relationship between age, sex, and race/ethnicity and fall outcomes (length of hospital stay (LOS), total medical charges) with linear regression models. In 2018, the rate of falls serious enough to warrant a hospital stay in Philadelphians aged 60+ was 243 per 10,000. This rate increased dramatically with age, from 116 per 10,000 (60-64) to 649 per 10,000 (85+). Men were at higher risk than women for each 5-year age group except those top-coded at 85+. Compared to white older adults, black older adults had greater risk at younger ages (60-69) and lower risk at older ages (70+). In linear models we found that charges and LOS decreased with increasing age. Both charges and LOS were higher for men than women. Hispanic patients had significantly higher charges than non-Hispanic patients, despite having similar lengths of stay. Future work will attempt to explain differences in charges and LOS by examining mortality, discharge location (e.g., home, hospice, rehab), and co-morbidities.

